# Collision Avoidance Mechanism for Swarms of Drones

**DOI:** 10.3390/s25041141

**Published:** 2025-02-13

**Authors:** Dariusz Marek, Piotr Biernacki, Jakub Szyguła, Adam Domański, Marcin Paszkuta, Marta Szczygieł, Marcel Król, Konrad Wojciechowski

**Affiliations:** 1 Department of Distributed Systems and Informatic Devices, Faculty of Automatic Control, Electronics and Computer Science, Silesian University of Technology, Akademicka 16, 44-100 Gliwice, Poland; dariusz.marek@polsl.pl (D.M.); adamd@polsl.pl (A.D.); marckro725@student.polsl.pl (M.K.); 2 Polish-Japanese Academy of Information Technology, Koszykowa 86, 02-008 Warsaw, Poland; pbiernacki@iitis.pl (P.B.); marcin.paszkuta@polsl.pl (M.P.); szczygielmarta@outlook.com (M.S.); konrad.wojciechowski@polsl.pl (K.W.); 3 Institute of Theoretical and Applied Informatics, Polish Academy of Sciences, Bałtycka 5, 44-100 Gliwice, Poland; 4 Department of Computer Graphics, Vision and Digital Systems, Faculty of Automatic Control, Electronics and Computer Science, Silesian University of Technology, Akademicka 16, 44-100 Gliwice, Poland

**Keywords:** swarm of drones, software-in-the-loop (SITL), hardware-in-the-loop (HITL), collision avoidance, positioning accuracy

## Abstract

This article presents a new approach to collision avoidance in drone swarms, designed for operations in large drone swarms and dynamic environments. The mechanism uses distributed communication, where drones share information about their positions and planned trajectories to predict and avoid collisions. The proposed mechanism enables drones to autonomously cooperate and maintain safe distances in complex scenarios. It is based on the concept of repulsion vectors. The avoidance response is determined by the level of immersion in the protective sphere of obstacles, including other drones. The advantage of the algorithm lies in its simplicity and low computational complexity, allowing it to be used even in small and inexpensive drones. The algorithm was tested in a developed simulation environment, created to handle swarms of over 20 drones and to demonstrate the scalability of the proposed solution. Two scenarios were analyzed: (i) two swarms, each with nine drones, flying on a collision course; (ii) a swarm of 25 drones changing formation. The results showed that the mechanism is effective in avoiding collisions, maintaining safe distances and adapting to changing conditions. The proposed mechanism represents a significant advancement in swarm coordination, offering a robust and scalable solution for real-world applications.

## 1. Introduction

The increasing possibilities for drone applications have led to technological development and the emergence of drone swarms. Drone swarms are groups of autonomous units that cooperate with each other to achieve a common goal [1]. Thanks to this cooperation, drones can perform more complex tasks that are beyond the capabilities of individual units [2,3]. In environmental monitoring, drone swarms are used to detect and track wildfires as well as monitor air and water quality. In precision agriculture, they enable crop condition analysis, irrigation control, and the detection of plant diseases. In rescue and crisis management, they deliver medical supplies to hard-to-reach areas and assist in the search for missing persons. In the entertainment industry, they are used in drone shows to create intricate formations and visual effects. In military applications, they are used for autonomous terrain reconnaissance and to support combat operations with minimal risk to manned units. The creation of mechanisms for drone groups that enable the execution of coordinated, complex maneuvers represents a major breakthrough in technology [4,5].

Swarm groups can carry out a wide range of tasks—from simple actions to advanced operations requiring autonomy. Autonomous drone technology requires complex control systems to reduce the risks and dangers associated with drone usage [6,7]. These systems use sensors, cameras, and topographic maps to effectively manage the formation [1,8].

One of the main challenges in controlling swarms is coordinating the swarm and avoiding collisions between drones. Achieving this goal requires drones to use advanced and computationally complex decision-making algorithms based on data from sensors [9,10].

There are many approaches to swarm control. These include nature-inspired algorithms that mimic the behaviors of social animals, such as birds, fishes, or bees [3]. Distributed consensus algorithms enable drones to reach a common state by exchanging information with their neighbors [9]. In leader-based models, one or more drones act as leaders, while the remaining units follow them [11]. The choice of control and management method depends on the specifics of the task, reliability requirements, hardware, and communication constraints. Obstacle avoidance algorithms in drone swarms, like in autonomous vehicles, use sensor data to analyze the environment dynamically [12]. This requires building a map of the surroundings. At the same time, the robot or vehicle’s location on the map must be determined using the SLAM (Simultaneous Localization and Mapping) technique [13]. Research also evaluates SLAM algorithm performance with different hardware setups by analyzing map accuracy [14].

This article presents a collision avoidance algorithm designed for specific swarms of drones. These swarms are intended to be based on a mesh communication system. This solution enables communication between all drones within the swarm and allows for the exchange of information about their positions and planned flight trajectories. The algorithm we propose achieves collision avoidance through continuous monitoring by all units of their positions relative to the swarm and the surrounding environment. A key feature of this algorithm is its low computational complexity. Thus, the implementation is feasible even for a swarm of drones consisting of many units. The developed mechanism enables collision avoidance with other drones in the swarm as well as with any static or dynamic objects in the drone’s vicinity.

Preliminary research on drone swarm control mechanisms and collision avoidance are conducted in a Software-in-the-Loop (SITL) simulation environment [15]. The correctness of the simulation results was confirmed through experiments on a real drone swarm. This approach enables faster software development and reduces operational risk [16,17]. The research focuses on the swarm dynamics in various scenarios, with particular emphasis on developing efficient collision avoidance mechanisms between drones and encountered objects. During the research, we also analyzed the impact of various swarm configurations on their coordination and collision avoidance capabilities under changing operational conditions. The implementation of the simulation environment, including the impact of communication delays and the accuracy of positioning systems, are presented in our previous articles [18,19].

The remainder of the paper is organized as follows: Section 2 describes the methods of controlling drone swarms, as well as the collision avoidance mechanisms for swarms of drones. Section 3 introduces our collision avoidance concept for a drone swarm and the theoretical assumptions of the proposed method. Section 4 presents the simulation environment and describes the results of the conducted experiments. Section 5 presents the conclusions drawn from the experiments and future work.

## 2. Drone Swarms and Collision Avoidance Mechanisms

The development of software and sensor technology enables drones to perform tasks autonomously, creating new possibilities for their applications [20]. With deep learning methods, drones can autonomously navigate to disaster sites or anomalies, enhancing their usefulness in emergency situations [21]. Additionally, simulation techniques enable rapid testing of swarm control strategies, which significantly supports their integration in industry [22].

In the literature, examples of effective distributed control methods can be found. One of such methods is swarm drone control based on distributed consensus algorithms. It enables the effective coordination of multiple units, drawing inspiration from animal behaviors [23]. Thanks to consensus algorithms, drones can quickly respond to obstacles and changes in the environment, enhancing their safety and stability in complex environments [10]. An example of such an algorithm is the leader model, where the movement of one drone serves as a reference for the others. The leader-based solution allows for the smooth management of swarm movement, enabling collision-free coordination, even at high cruising speeds and with significant communication delays.

In research on drone swarms, collision avoidance is a critical issue. Many units in the swarm must maintain an appropriate distance to avoid violating the safety zone. Many units in the swarm must maintain the proper distance to avoid encroaching on the safety zone [11,24]. The method presented in this article assumes that each drone is capable of autonomously detecting and avoiding obstacles, as well as coordinating its movements with other units. The work [25] provides an overview of various sensor technologies and detection methods used in UAV anti-collision systems. The authors present a comprehensive analysis of sensors such as radar, ultrasound, and optics in terms of their applications for obstacle detection and collision avoidance. Radar systems are particularly highlighted for their ability to operate in low-visibility conditions. Adaptive obstacle avoidance algorithms, which enhance flight safety, are also described. The article [26] highlights the advantages of distributed control based on graph theory that enables us to model the communication between drones in a swarm network. This enhances the swarm’s ability to respond to obstacles and increases the precision of maintaining formation in dynamic and changing environments. In the literature [27], a collision avoidance algorithm for unmanned surface vehicles (USVs) is also presented, which is named the optimal collision avoidance point (OCAP). The algorithm determines a route to avoid static and dynamic obstacles in real time. OCAP calculates the optimal collision avoidance point based on relative velocities and kinematic parameters. It then assesses the collision risk for various trajectories. Research has shown that OCAP is efficient and reliable in high-density obstacle environments compared to traditional methods, such as the Dynamic Window Approach (DWA). The authors of the article [28] developed a collision avoidance system based on fuzzy inference and variable space search. The tests presented in the article confirmed its effectiveness in head-on and crossing maneuvers. The work [29] presents the application of artificial intelligence techniques in obstacle detection. The Adaptive Collision Avoidance Algorithm Based on the Estimated Collision Time (ACACT) proposed in the article uses the estimated time to collision (when the drone could potentially collide with an obstacle) to dynamically adjust the trajectory. In unmanned aerial vehicle (UAV) systems, minimizing the impact of the Age of Information (AoI), which determines the timeliness of data transmitted from drones to the base station, is also crucial. In the work [30], the problem of minimizing the long-term average AoI in a UAV-assisted Wireless Powered Communication Network (WPCN) was investigated. In this system, the UAV plays a dual role: (1) as a mobile data relay and (2) as an energy source for sensors deployed on islands. The drone’s battery has limited capacity, necessitating the development of a strategy for efficient island clustering and flight trajectory optimization.

The authors proposed a Hybrid TDMA-NOMA Protocol (HTN), which dynamically adjusts the transmission method based on the energy levels in the sensors. The algorithm considers flight distances and the number of sensors to optimize energy consumption. Research has shown that this mechanism effectively minimizes AoI, thereby reducing the number of returns to the base. This solution can be applied in environmental monitoring and remote control systems. However, the method has its limitations. The main challenge is synchronization between sensors and the UAV, which leads to transmission delays. This is particularly problematic when dealing with a large number of devices. Meanwhile, in the work [31], a method for determining and optimizing UAV trajectories was proposed, where drones dynamically adjust their routes to changing environmental conditions. The research results demonstrated that algorithms such as Multi-Agent Deep Reinforcement Learning (MADRL) can effectively synchronize unit movement in airspace and prevent collisions. Despite its effectiveness, this solution also has certain limitations. Machine learning- or deep learning-based models [31] may require significant computational power. For this reason, these solutions are less practical for real-time applications such as demonstration drones.

In the work [32], the authors conducted research on the problem of UAV localization in areas where GPS is unavailable. They proposed an enhanced version of the Particle Swarm Optimization (PSO) algorithm to determine the drone’s position. To improve accuracy and reduce computational load, they introduced two new methods: Hierarchical PSO (HPSO) and Reference PSO (RPSO). However, these methods may face challenges in dynamic environments and require substantial computational resources.

The work [33] presents the Multi-drone Sensing Experimentation Testbed (M-SET) platform, featuring a collision avoidance mechanism based on potential fields and a collective multi-agent learning method. The applied potential field algorithm generates attractive and repulsive forces that guide the drones toward their targets while avoiding collisions with other drones and static obstacles. The authors [34] developed a UAV swarm path planning and collision avoidance system based on the Artificial Bee Colony (ABC) algorithm. Inspired by bee behavior, the algorithm optimizes flight speed and minimizes the distance to the target while simultaneously avoiding collisions. Simulation studies involving 12 and 20 drones demonstrated effective collision avoidance. However, with 50 drones, 12 potential collisions were observed. The article [35] describes a decentralized collision avoidance algorithm in 3D environments. The system uses predictive models to calculate potential collision trajectories and accordingly adjusts the movement of the units, taking into account communication delays and sensor limitations. The authors [36] proposed an algorithm that uses lidar data for precise real-time mapping of the environment. Based on these data, the system develops trajectories for avoiding both dynamic and static obstacles, such as buildings or other UAVs. The algorithm dynamically updates the drone’s path to minimize the risk of collision. A path planning strategy and collision risk management in multi-drone systems is presented in [37]. The algorithm uses 3D mapping and predicts the movement of other UAVs to avoid potential collisions. Special attention is given to priority management in traffic conflicts. This allows for effective collision risk management when multiple drones move simultaneously within a swarm. The method presented in the article assumes that each drone is capable of autonomously detecting and avoiding obstacles, as well as coordinating its movements with the other units [11,24]. In the scientific literature, attempts have been made to solve the collision avoidance problem in UAV swarms using a leader–follower model [38]. The mechanism described in [39] is based on local obstacle avoidance using a lidar, while the drones in the formation follow the leader. Each sector is evaluated in terms of safety. The UAV then selects the safest direction. This is a local approach to obstacle avoidance. This approach is effective in scenarios with static obstacles.

Table 1 presents a general summary of the key concepts in drone swarm control.

The collision avoidance mechanism described in this article is based on a distributed consensus system and continuous exchange of information between drones regarding their positions and planned flight trajectories. It is based on the concept of repulsion vectors. The avoidance response is determined by the level of immersion in the protective sphere of obstacles, including other drones. The accuracy and efficiency of the proposed algorithm were demonstrated in simulation studies and in real-world tests with a swarm of drones. For research purposes, a specialized distributed simulation model was proposed, allowing for simulation studies of very large swarms of drones (more than 20 units).

## 3. The Concept of a Collision Avoidance Mechanism for a Drone Swarm

In our concept of a collision avoidance mechanism for drone swarms, the key element is the architecture of the system based on distributed communication between units. Each drone in the swarm is equipped with a set of sensors, such as a compass, IMU, and barometer, as well as an RTK GPS module, providing precise positioning with an accuracy of up to 5 cm [40].

**Figure 1:**
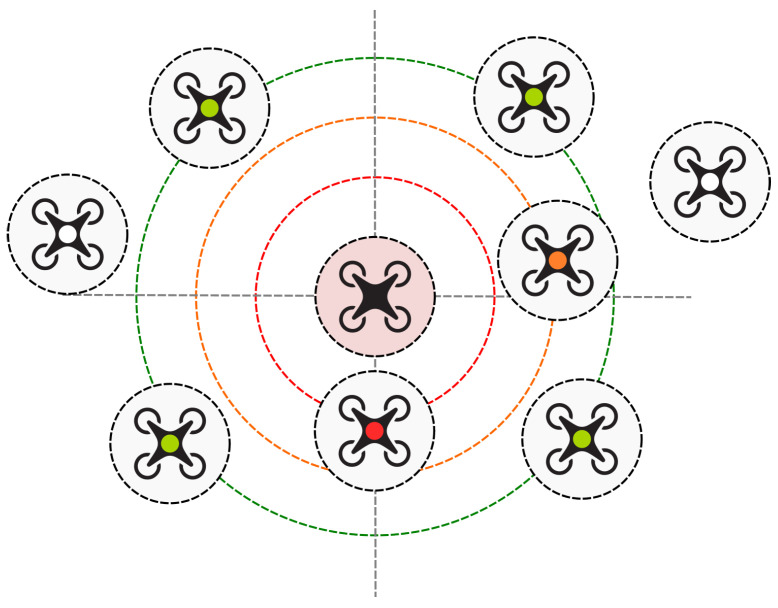
Schematic diagram illustrating the collision avoidance mechanism.

Figure 1 illustrates a conceptual model of the collision avoidance mechanism for drone swarms. Units in the swarm constantly monitor their position relative to the other drones. The collision avoidance mechanism ensures safe distances between the drones, which minimizes the risk of collisions and enables precise synchronization of drone actions. The system operates autonomously, ensuring continuous coordination of the drone swarm’s movement in a dynamically changing environment. The drones communicate directly with each other using a mesh network topology, employing the MAVLink protocol to exchange information, such as locations and planned routes. Our studies on 20 real communication modules connected in a mesh network showed that such transmission occurs with acceptable delays and no packet loss.

The system architecture supports scalability, allowing for the easy addition of new drones to the swarm. The failure of individual units does not interrupt the execution of the mission. The remaining operational drones can continue the mission even if the number of drones in the swarm is reduced. Communication efficiency is ensured by minimizing delays and ensuring reliable data exchange, which is crucial for maintaining small distances between drones and optimizing mission effectiveness.

During flight, data are continuously collected and used to dynamically generate an environmental map, which is shared among all units. Based on this map, drones adjust their routes to effectively avoid obstacles and prevent collisions. This system ensures efficient coordination even in densely populated areas or challenging terrains.

### 3.1. Drone Motion Model

The movement of any drone in three-dimensional space can be described using a simplified kinematic model. Instead of expressing position and velocity as continuous functions of time (as in the case of solving motion problems using differential equations), motion primitives are used [41,42].

Each motion primitive defines a short trajectory generated by a control signal, forming a finite set of possible trajectories. This method leverages the differential flatness of quadcopters, allowing the position trajectory to be approximated with a polynomial function [41]:

(1) $$p\left(t\right)=c_{k}t^{k}+\ldots +c_{1}t+c_{0}\in R^{3}$$

where $c_{k}$ are polynomial coefficients and *k* denotes the polynomial order.

Motion primitives simplify the description of trajectories in complex environments by discretizing the state space into a grid. Each trajectory segment accounts for control constraints, such as maximum speed and acceleration, ensuring feasibility [41].

### 3.2. Trajectory Optimization

Collision avoidance and trajectory optimization are handled in a discrete structure. Instead of solving continuous equations, trajectory planning is modeled as a graph search problem [42]. The cost function is designed to minimize collision risk while maintaining the planned trajectory:

(2) $$J\left(x\right)=\sum_{i=1}^{N}\sum_{j\in N_{i}}\left(\frac{1}{∥p_{i}-p_{j}{∥}^{2}}\right)+\lambda {∥v_{i}-v_{d}∥}^{2}$$

where $p_{i}$ and $p_{j}$ represent drone positions, $v_{d}$ is the desired velocity, and λ balances collision avoidance and trajectory adherence, where each node represents a state, and the edges correspond to feasible motion primitives [41,42]. The cost function (Equation (2)) has been designed to minimize the risk of collision and increase the probability of keeping the drone as close as possible to the planned trajectory. The first term of the function aims to increase the distance between drones, which reduces the likelihood of a collision. The second term ensures drones stay close to the designated trajectory at the desired speed. This balance enhances both safety and mission efficiency.

### 3.3. Generating Motion Primitives

Generating motion primitives involves computing discrete trajectory segments using predefined control signals within a specified time interval Δt [41,42]. For each segment, position and velocity are updated iteratively as follows:

(3) p(t+Δt)=p(t)+v(t)Δt,v(t+Δt)=v(t)+u(t)Δt

where u(t) is the control vector (acceleration). The iterative process replaces continuous differential equations, making it suitable for real-time applications in complex environments [42].

### 3.4. Collision Avoidance Prediction

Drones belong to the group of dynamically moving objects. The collision avoidance algorithm is activated only when the minimum safety distance $d_{\min}$ has been breached. This approach may not be sufficient when drones travel at high speeds and are on a collision course, such as a head-on or perpendicular collision. The solution to this problem is to predict potential collision points with other drones in the swarm and adjust their speed or stop at a safe distance from the predicted collision position.

Through real-time information exchange between drones regarding their positions, each drone is able to determine the trajectory and speed of other drones in the swarm. By knowing the trajectories and speeds of other drones, it becomes possible to estimate their future positions and predict the potential location of the earliest collision.

The algorithm analyzes the future positions of drones over successive time steps $t_{\mathrm{iter}}$ and checks whether the distance between the future position of the analyzing drone $p_{i}\left(t+t_{\mathrm{iter}}\right)$ and the position of another drone in the swarm or an obstacle $p_{j}\left(t+t_{\mathrm{iter}}\right)$ is not smaller than the defined minimum safe distance $d_{\min}$. If the boundary of this distance is breached, the analyzing drone calculates the distance to the potential collision position $d_{\mathrm{col}}$ and adjusts its position to maintain a safe distance, while staying on course toward its destination.

(4) $$p_{\mathrm{safe}}\left(t\right)=p_{i}\left(t\right)+{\hat{v}}_{d}\left(d_{\mathrm{col}}-d_{\min}\right)$$

The calculated position $p_{\mathrm{safe}}\left(t\right)$ is used to compute the control velocities of the drone.

### 3.5. Calculating the Desired Trajectory

The presented algorithm for estimating the desired drone trajectory involves determining the resultant force of the repulsive vectors exerted on the analyzing drone by other swarm units and environmental elements. The calculated resultant vector also takes into account the need to reach a designated destination.

To ensure smooth collision avoidance, an innovative weighting method has been applied, modifying the repulsive force vector in relation to a given obstacle. It also allows for balancing opposing forces from multiple sources and determining the desired position between multiple obstacles or drones exerting pressure on the analyzing unit.

If the distance between the drone and an obstacle is smaller than the minimum safety distance, the repulsive vector *r* relative to the given obstacle is calculated as follows:

(5) $$r\left(t\right)=p_{i}\left(t\right)-p_{j}\left(t\right)$$

and a weight modifying its strength is calculated as follows:

(6) $$\beta =\frac{1-\min\left(1,\frac{∥r(t)\Delta t∥}{d_{\min}}\right)}{1-\mathrm{urr}}$$

where urr∈[0.5,1) is a parameter that divides the drone’s safety zone into two regions: an inner and an outer zone.

By using the parameter urr, it is possible to define the degree of softness of the collision avoidance system’s response to an encountered obstacle. This parameter ensures the weight function remains nonlinear across the entire safety zone while maintaining linearity in both the inner and outer regions. The computed weight function smoothly transitions between 0 and 1 within the safety zone boundaries. Once the obstacle breaches the inner safety threshold, the weight value increases, reaching a maximum of 2.

The final position-modifying vector for the analyzing drone is the resultant vector R(t), which considers the repulsive vectors $r_{j}\left(t\right)$ along with the weights $\beta_{j}$ and the attractive vector $v_{d}\left(t\right)$ which represents the movement toward the target:

(7) $$R\left(t\right)=\sum_{j=1}^{N_{i}}\left({\hat{r}}_{j}\left(t\right)\beta_{j}\right)+\lambda v_{d}\left(t\right)$$

The implemented Algorithm 1 is an integral component of the drone’s flight trajectory determination mechanism. Each drone in the swarm has real-time access to the position of all other nodes, enabled through continuous data exchange using the RTK GPS system. The mechanism also facilitates the detection of unexpected objects in the swarm’s airspace. In future system iterations, additional sensor data, such as lidar readings, will be incorporated. This enhancement will further refine precision and improve flight path adaptation to dynamic environmental conditions.
**Algorithm 1** Drone control algorithm.1:Initialize the parameters $d_{\min},\mathrm{urr},\lambda$2:**while** the drone has not reached the target **do**3:   **for** each position $p_{j}$ (drones and obstacles) **do**4:     Calculate the repulsive vector $r_{j}\left(t\right)=p_{i}\left(t\right)-p_{j}\left(t\right)$5:     **if** $∥r_{j}\left(t\right)\Delta t∥<d_{\min}$ **then**6:        Calculate the weight $\beta_{j}$7:     **end if**8:   **end for**9:   Calculate the resulting vector:$$R\left(t\right)=\sum_{j=1}^{N_{i}}\left({\hat{r}}_{j}\left(t\right)\beta_{j}\right)+\lambda v_{d}\left(t\right)$$10:   Update the drone’s position:$$p_{i}\left(t+\Delta t\right)=p_{i}\left(t\right)+R\left(t\right)\Delta t$$11:**end while**

## 4. Simulation Experiments

To evaluate the effectiveness of our collision avoidance algorithm, we conducted a series of simulations in a simulation environment based on the AirSim platform [43]. The simulation environment was developed using the official source code and Unreal Engine. A basic 3D terrain was designed to provide an obstacle-free space for the swarm. The physical prototypes were built utilizing the Holybro X500 frame, Pixhawk 6C flight controller, 2216 KV920 motors, 1045 propellers, a 4S 5000 mAh battery, and custom 3D-printed components, including the chassis, fastening elements, and protective covers. Although various drone models are available in the AirSim environment, none precisely match our physical prototype. In AirSim, model parameters are defined in the source code, making it impossible to modify the physical characteristics of the kinematic model after compilation. To overcome this limitation, we transferred the kinematic model parameters to a configuration file, allowing for effortless modifications without requiring recompilation. The assumptions underlying the simulation environment were established in our previous work, as presented in [18,19].

The drone model was adapted to real-world physical parameters, such as mass, dimensions, and the characteristics of the motors and batteries. The simulation environment allowed for testing the algorithms under controlled conditions, eliminating the risk of damaging real drones.

The primary objective of the conducted simulations was to evaluate the effectiveness of the developed collision avoidance mechanism under various operational conditions. The studies considered variables such as flight speed, obstacle density and distribution, and dynamic environmental changes.

Two specific scenarios were developed as part of the research. In Scenario I, the study was conducted for 18 drones, divided into two swarms of nine drones each. Each swarm was arranged in a 3 × 3 grid formation, with a leader positioned in the center of the grid, followed by the remaining drones. The distance between drones was set to 5 m, and the drones’ speed reached up to 5 m/s. The leaders were placed 200 m apart, and a collision course between the two swarms was simulated. Due to the assumption that the drones would fly in formation behind the leader, the collision avoidance mechanism’s behavior was most visible in the analysis of the distance between the leaders.

In Scenario II, the study was conducted for a swarm of 25 drones in a grid formation. The distance between drones was set to 5 m and 20 m, with the drones’ speed once again reaching up to 5 m/s. The study verified the operation of the collision avoidance mechanism as the drones transitioned from the initial formation to the final formation. The flight trajectories of 24 drones intersected at the center of the grid, requiring a change in their trajectories in 3D space. The leader’s position remained unchanged. The experiments were planned in such a way as to result in the maximum number of potential collision paths between drones, allowing for an evaluation of the effectiveness of the developed mechanism. It was assumed that a collision would occur if the distance between drones became less than or equal to 1 m. This value was determined based on the actual size of the drone and the span of its propellers.

All experiments were repeated multiple times. We present detailed results for some of the worst-case scenarios (where the smallest distances were observed). It should be emphasized that no collisions occurred in any of the experiments.

### 4.1. Scenario I

**Figure 2:**
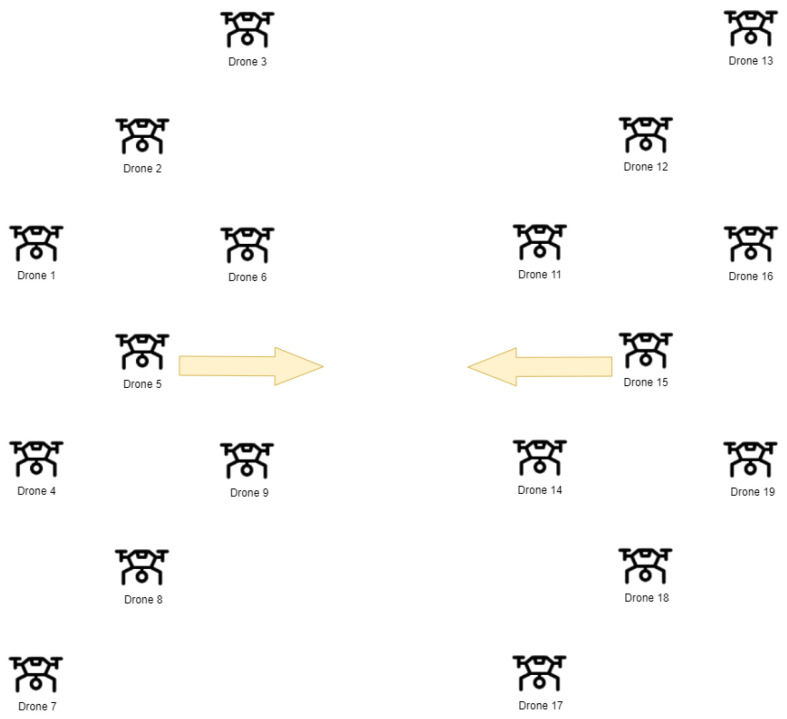
The visualization of the initial state of drones.

**Figure 3:**
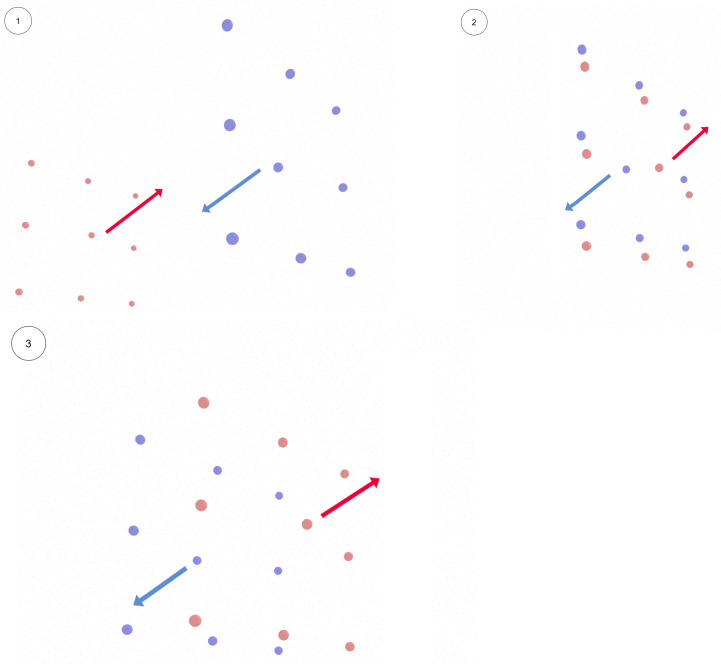
Change in the formation’s position during mission execution (1—the state before the start, 2—before the collision, 3—during movement along the collision course).

Figure 2 illustrates the initial drone positions in Scenario I. The arrows indicate the directions of swarm movements. In Figure 3, a sequence of the drone formations movement is shown (two swarms approach each other on the collision course). Drones belonging to the first swarm are marked in blue, drones belonging to the second are marked in red and the arrows indicates directions of swarm movements.

As part of the conducted research, a simulation was developed involving two swarms, each consisting of nine drones, which were directed onto a collision course while maintaining an initial distance of 5 m between the units and a maximum flight speed of 5 m/s. The parameter urr, which defines the intensity of the collision avoidance reaction, took the values of 0.5, 0.75, and 0.99. The parameter urr = 0.5 means the maximally delayed reaction, whereas urr = 0.99 corresponds to a very early response of the drones to approaching each other.

**Figure 4:**
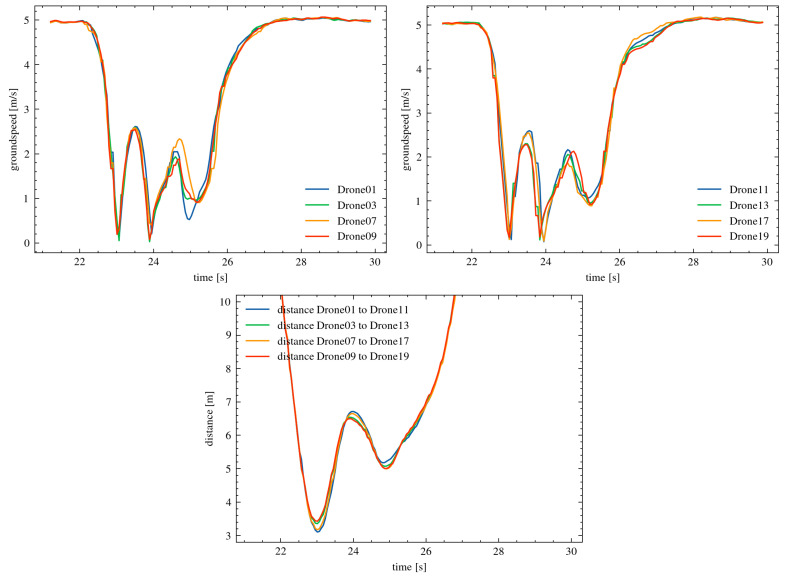
The speed chart of selected drones for both swarms ((**left**) and (**right**) charts) and the minimum distances ((**middle**) chart) for the parameter urr = 0.75.

The simulation results showed that the developed collision avoidance mechanism effectively prevented collisions in all tested cases, regardless of the urr parameter value. In Figure 4, the changes in the speeds of individual drones and the minimum distances between them during the experiment are presented.

For a parameter value of urr = 0.5, the minimum distance between drones decreased to 1.686 m. This distance shows that the mechanism’s response was delayed, but it still managed to avoid a collision. For urr = 0.75, the minimum distance was 1.975 m, indicating a more balanced and quicker response from the drones. In the case of urr = 0.99, where the response was very early, the minimum distance increased to 2.621 m. These results confirm the effectiveness of the algorithm in maintaining a safe distance. The simulation results confirm that the developed collision avoidance mechanism is effective and scalable. In the experiments, the drones moved along collision courses, meaning they were directly directed at each other. The mechanism dynamically adjusted the intensity of the drones’ reactions, preventing collisions even in challenging conditions. The results show that regardless of the set parameter urr, the minimum distance between the drones was sufficient to avoid a collision. The detailed changes in speed and distance presented in the charts confirm the effectiveness of the solution. The values of the minimum distances between individual drones, which were on collision courses, are provided in the Table 2.

**Figure 5:**
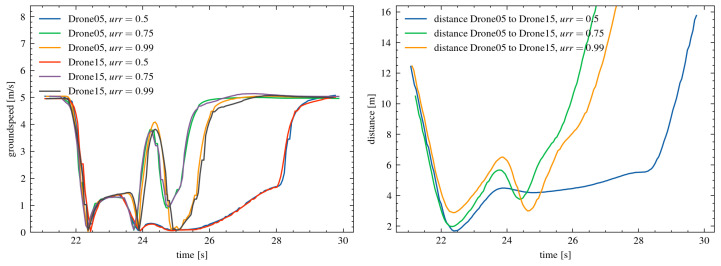
The speed of the leader drones (**left**) and the distance between the leader drones (**right**) for different values of the parameter urr = 0.5, 0.75, and 0.99.

In the charts (Figure 5), the changes in distance (left chart) and speed (right chart) for the leader drones (Drone05 for the first swarm and Drone15 for the second swarm) are presented for three values of the parameter urr = 0.5, 0.75, and 0.99.

For a parameter value of urr = 0.5, the distance between the leader drones initially drops very sharply, reaching a minimum of 1.69 m. Afterward, the distance increases more slowly compared to the other urr values. The drones’ responses are delayed here, resulting in the smallest safety margin. For urr = 0.75, the minimum distance is greater than for urr = 0.5. The response to approaching the object occurs much faster. This allows for a smoother response to a potential collision.

In the case of urr = 0.99, the drones respond the earliest. This is evident in the rapid increase in distance after reaching the minimum. The minimum distance is 2.88 m. The flight trajectory and the collision avoidance mechanism’s response are the smoothest. The analysis of the observed speeds of the leader drones indicates significant speed reductions in the moment of collision avoidance for urr = 0.5. The mechanism’s response is delayed, causing large changes in speed. For urr = 0.75, the drones’ speeds also decrease, but their dynamics are more controlled. Urr = 0.99 affects the fastest response, which helps avoid sudden speed drops. The leader drones’ speeds remain more stable. The obtained results indicate that the parameter urr significantly affects the effectiveness of collision avoidance. For urr = 0.99, the drones’ responses are the fastest. This allows for maintaining a greater minimum distance and more stable speeds. On the other hand, lower values of urr (0.5) lead to delayed responses. This results in a smaller safety margin and larger speed fluctuations. The values of urr = 0.75 represent a compromise between stability and speed of response.

### 4.2. Scenario II

**Figure 6:**
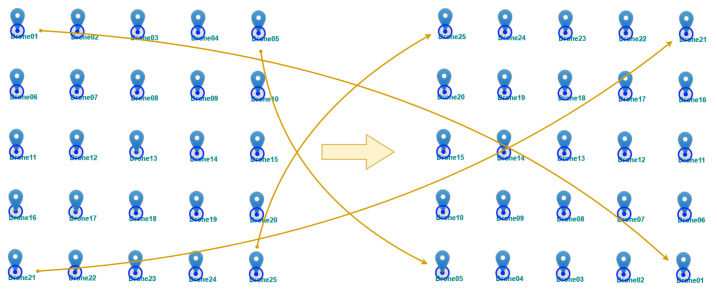
The concept of changing the position of drones in a swarm by 180 degrees relative to the initial position.

**Figure 7:**
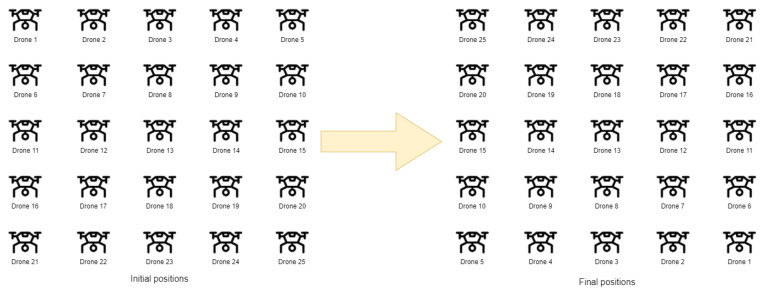
The geographic distribution and mobility model of drones.

Figure 6 presents the schematic concept of changing the position of all drones in a swarm by 180 degrees. Figure 7 introduces the geographic distribution and mobility model of drones. The arrows indicate the movement from the initial to final position of the swarm.

In the first phase of the experiments in Scenario II, the distance between the drones was set to 20 m. The value of the parameter urr, which determines the intensity of the drones’ repulsion in order to maintain a safe distance, was set to 0.75 (a value of 0.99 corresponds to an early reaction, while 0.5 corresponds to a maximally delayed reaction).

**Figure 8:**
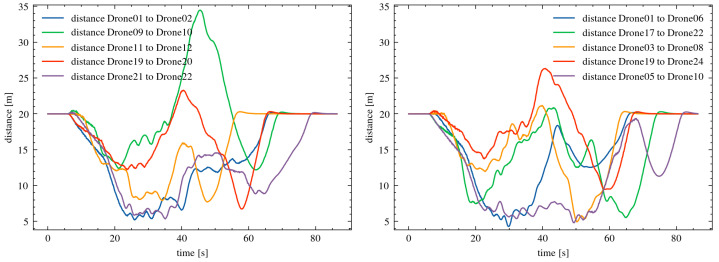
Changes in the distance between neighboring drones in the formation, for an initial distance of 20 m between the drones.

Figure 8 presents detailed changes in the distance between neighboring drones during the course of the experiment. The developed mechanism enabled the effective management of the drones’ flight in a scenario with obstacles moving at speeds of up to 5.0 m/s. The maintained distances from obstacles were at least 2.647 m. These results demonstrate the effectiveness of the algorithm in handling dynamic environments with moving objects within the drone swarm’s flight area.

**Figure 9:**
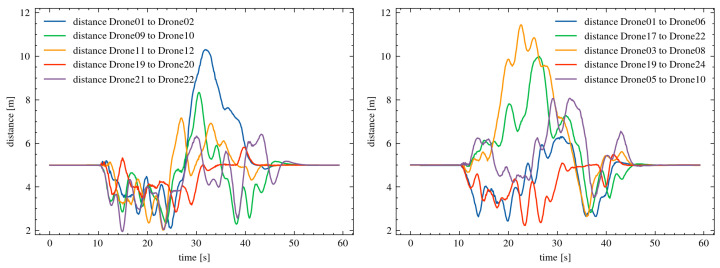
Changes in the distances between neighboring drones in the formation, with an initial distance of 5 m between drones.

In the next stage of the research, simulations were conducted with 25 drones moving in close proximity. The distance between the drones was reduced to 5 m. The results of the experiments showed that the developed mechanism successfully prevented collisions during the mission execution. The minimum distance between the drones was 1.396 m. Figure 9 presents detailed changes in the distances between adjacent drones throughout the experiment. The results of all experiments demonstrate the scalability of the developed algorithm. The mechanism ensured proper management of collision paths for drones across different types of missions, variable swarm sizes, and varying initial distances between the drones.

The conducted simulations confirm the effectiveness of the developed algorithm under various spatial and dynamic conditions. The simulation results provide a solid foundation for further experiments in real-world conditions.

## 5. Conclusions

Drone swarms can be utilized in various applications, ranging from monitoring to rescue operations. The conducted experiments open up new possibilities for their use in many emerging fields. One such application could be swarms dedicated to drone shows. Currently, existing solutions rely on individually setting flight trajectories for each drone. The proposed solution in this article, featuring a leader drone and a collision avoidance mechanism, significantly simplifies the process of preparing such a show.

This article proposes a novel collision avoidance mechanism. The developed algorithm was implemented and its effectiveness was verified using simulation methods. Two mission scenarios were designed for this study. The scenarios are characterized by a high number of flights on collision trajectories. The results of the experiments unequivocally confirmed the effectiveness of the mechanism in adapting to dynamically changing operational conditions. They demonstrate that the developed mechanism maintains appropriate distances between the drones and static objects.

The use of advanced simulation tools significantly facilitates the study of the collision avoidance system for drones moving in a swarm and taking into account operational and environmental conditions such as speed, formation changes, and obstacle avoidance.

Implementing new solutions is crucial for ensuring the safety and efficiency of drone swarm operations, especially in situations that require rapid response and precise maneuvering, as well as in cases of communication interruptions or delays. The low computational complexity allows for implementation in small and cost-effective drones.

Drone swarms can be utilized in a variety of applications, from monitoring to rescue operations. These experiments can open new possibilities for their application in various fields.

The collision avoidance mechanism effectively prevented collisions in all conducted tests, regardless of the parameter urr:

- With urr=0.5, the algorithm exhibited a delayed response, resulting in a minimum distance of 1.69 m between the drones. Despite this, the collision avoidance mechanism worked correctly.
- For urr=0.99, the reaction was fastest, ensuring larger minimum distances (up to 2.88 m) and smoother trajectory.
- The value of urr=0.75 represented the best compromise between reaction speed and stability, which was evident both in the minimum distances and the dynamics of the drones’ movement.

The algorithm worked effectively in scenarios with varying initial distances between the drones (ranging from 20 m to 5 m). Even for demanding flight scenarios, the minimum distance between drones was 1.396 m, which confirmed the correctness of the algorithm.

The mechanism ensured stability in the drones’ speeds, minimizing sudden changes. This was particularly noticeable with urr=0.99, where the system responded the fastest while maintaining stable trajectories and speeds.

Our research unveils several critical insights:

- We proposed an algorithm with relatively low computational complexity, making it suitable for use in small and inexpensive drones.
- The proposed algorithm is based on continuous communication between drones and distributes the collision avoidance problem across all nodes in the swarm. Our mechanism is adapted to dynamic changes in the environment.
- The parameterizable approach allows for active regulation of the swarm’s operation.

In future work, we plan to conduct experiments with the developed mechanisms in real-world conditions. We also anticipate further development of the mechanisms by integrating additional sensors, such as thermal cameras or multispectral cameras. This will allow for even more precise obstacle detection and enhance the safety of drone swarm missions.

## Figures and Tables

**Table 1:** Comparison of drone swarm control methods.

Method	Advantages	Disadvantages
Nature-inspired algorithms	- Scalable for large swarms. - Simple rules inspired by animal behaviors [3].	- Limited precision in tasks requiring high accuracy. - Adaptation difficulties in dynamic environments.
Consensus algorithms	- Decision decentralization. - Stability in dynamic conditions [9].	- High communication requirements. - Complexity of implementation in large swarms.
Leader-based models	- Simplicity of coordination. - Effective in structured tasks [11].	- Sensitivity to leader failure. - Limited autonomy of the remaining units.
Deep learning algorithms	- Enable autonomous navigation and obstacle detection [21]. - High efficiency in complex environments.	- Require high computational power and energy. - High cost and difficulty in implementing new behavior rules.
Trajectory optimization (path planning)	- Efficient swarm movement management. - Minimizing the risk of collisions [37].	- High computational complexity for dynamic environments. - Sensitivity to delays in communication systems.
Potential fields	- Simple to implement for collision avoidance [33]. - Natural adaptation to the environment.	- Possibility of generating dead zones. - Problems with maintaining stability in dense formations.
Proposed mechanism—a method based on the concept of repulsion vectors. The avoidance response is determined by the level of immersion in the protective sphere of obstacles, including other drones.	- Low computational requirements, suitable for large swarms. - Avoidance of static and dynamic obstacles. - Use of mesh communication for exchanging position and trajectory data [15]. - Ability to parameterize the intensity of collision avoidance.	- Requires data synchronization with high frequency.

**Table 2:** The minimum distances between drones [m] for urr = 0.5, 0.75, and 0.99.

The Distances Between Drones	urr = 0.5	urr = 0.75	urr = 0.99
Drone01—Drone11	3.0740	3.1085	4.2240
Drone02—Drone12	3.2801	3.5116	4.1203
Drone03—Drone13	3.0168	3.3540	4.1241
Drone04—Drone14	2.8889	3.3520	4.2383
Drone05—Drone15	1.6863	1.9759	2.8827
Drone06—Drone16	3.1735	3.0667	4.0470
Drone07—Drone17	3.2019	3.1786	3.9095
Drone08—Drone18	3.1028	3.2995	4.0981
Drone09—Drone19	3.0241	3.4265	4.0682

## Data Availability

The raw data supporting the conclusions of this article will be made available by the authors on request.
